# Effective Reinforcement of Visible Light Photocatalytic and Gas Sensing Characteristics of Nanocrystalline TiO_2_: Gd-Based Nb and Mo Dopants

**DOI:** 10.3390/molecules28217239

**Published:** 2023-10-24

**Authors:** Ghayah M. Alsulaim

**Affiliations:** Department of Chemistry, Faculty of Science, King Faisal University, Al Ahsa 31982, Saudi Arabia; g.m.alsulaim@gmail.com or galsaleem@kfu.edu.sa

**Keywords:** titanium dioxide, new compositions, environmental pollution, dye waste, photocatalysis, gas sensing properties

## Abstract

Efficient compositions for the selective detection of ethanol gas and the removal of organic contaminants were realized by codoping of (Gd, Nb) and (Gd, Mo) ions into TiO_2_. TiO_2_, Ti_0.96_Gd_0.01_Nb_0.03_O_2_, and Ti_0.96_Gd_0.01_Mo_0.03_O_2_ samples were prepared by a coprecipitation method. For all compositions, a crystalline anatase phase of TiO_2_ was detected. Compared to pure TiO_2_, the absorption edges of Ti_0.96_Gd_0.01_Nb_0.03_O_2_ and Ti_0.96_Gd_0.01_Mo_0.03_O_2_ samples were red-shifted, further broadening towards visible light. The morphological studies demonstrate that the grains of TiO_2_ were more refined after (Gd, Nb) and (Gd, Mo) codoping. The photocatalytic efficiency of the Ti_0.96_Gd_0.01_Mo_0.03_O_2_ catalyst for degrading 20 mg/L reactive yellow 145, brilliant green, and amoxicillin was 98, 95, and 93% in 90 min, respectively. The reusability experiments indicate that the Ti_0.96_Gd_0.01_Mo_0.03_O_2_ catalyst had high stability during reuse. The high photocatalytic activity of the Ti_0.96_Gd_0.01_Mo_0.03_O_2_ catalyst was correlated to the broad visible-light absorption and effective separation of electron–hole pairs by Gd^3+^ and Mo^6+^ cations. The gas sensing characteristic is reflected by the high sensitivity of the Ti_0.96_Gd_0.01_Nb_0.03_O_2_ sensor to ethanol gas in the presence of different gases at 275 °C. The obtained results indicated that the (Gd, Mo) mixture could more effectively induce the photocatalytic properties of TiO_2_ while (Gd, Nb) dopants were the best for reinforcing its sensing characteristics.

## 1. Introduction

In recent years, the monitoring of harmful gases and the treatment of waste organic dyes and antibiotics compounds by green sustainable techniques have emerged as important issues for the scientific community [1,2,3,4]. Organic dyes are compounds used to color a substrate like cloth, leather, paper, plastic, wool, polymers, nylon, and many others [5]. Technically, organic dyes improve the visual value of these products, which has broadened their use in almost all basic products. Unfortunately, colored organic dyes have strong adverse effects on the environment and human, animals, birds, plants, and aquatic life, even at low concentrations [5,6]. These colored organic dyes are soluble in water and many other organic solvents, chemically stable, and non-biodegradable; in addition, they are biologically active with carcinogenic and mutagenic characteristics [5,6,7]. Every year, 7 × 10^5^ tons of organic dyes are produced and nearly 200,000 tons of them, as textile dyes, are lost annually in effluent as wastewater [8]. Additionally, the remaining antibiotic pollutants in the aquatic system area a dangerous health issue owing to the emergence of antibiotic-resistant bacteria and their disruption of environmental stability [9].

Photocatalysis based on sunlight energy is a promising and green approach to eliminating these pollutants and realizing a clean and health environment [10,11]. Solar energy contains approximately 52% infrared (700–2500 nm), 43% visible light (400–700 nm), and 5% ultraviolet (280–400 nm) radiation [12]. The photocatalytic degradation of organic contaminants has many benefits over traditional techniques, including less energy consumption, better reusability, economic potential, and the non-harmful products [13]. There is a great demand for innovative, operative, inexpensive, and wide-responsive photocatalysts for wastewater treatment [14,15]. Numerous metal oxides, such as TiO_2_ [16], ZnO [17], CuO [18], and SnO_2_ [19], have been investigated for the photocatalytic degradation of organic pollutants. Among these metal oxides, titanium dioxide (TiO_2_) has emerged as a potential photocatalyst for degradation of various organic dyes [20,21]. Enhancing the photocatalytic properties of the TiO_2_ semiconductor is mainly achieved through two points: reducing the recombination of the charge carriers and prolonging its absorption in the visible light spectrum [22,23]. Many strategies have been used for this purpose: nanocomposite or heterostructures formation [23], doping [22], and codoping [24]. Chobba et al. [25] studied the photocatalytic activity of Gd-doped TiO_2_ for 25 mg/L methylene blue degradation under UV and visible light radiation. They found that 1% Gd-doped TiO_2_ has a degradation efficiency of 80% but only after 5 h of UV irradiation. Gd-doped TiO_2_ nanotubes displayed a photocatalytic activity of 95.8% for methyl orange (MO) dye in an irradiation time of 120 min [26]. Xu et al. [27] reported that Gd-doped TiO_2_ catalyst synthesized by the sol–gel method had a photodegradation efficiency of 65.6% and 54.2% for Reactive Brilliant Red X-3B and rhodamine B after an irradiation time of 80 min, respectively. Under UV irradiation, 0.5% Gd-doped TiO_2_ catalyst (hydrothermal route) exhibited a photodegradation activity of 92% for Acriflavine dye after 120 min [28]. Thus, using the sunlight as a source of radiation to increase the photocatalytic efficiency as well as reduce the reaction time is highly required for practical applications.

The detection, discrimination, controlling, and measuring of different types of gases are very important in numerous fields, such as food industry production [29], medical diagnosis applications [30], chemical warfare [31], detection of explosive, toxic, and flammable compounds [32,33,34], and traffic accidents [35]. Various industries and laboratories activities release many volatile organic compounds (VOCs), like acetone, butanol, ethanol, benzene, methanol, propanol, xylene, and toluene, to the environment and these gases are dangerous to human health and can cause deaths [36,37]. Among the volatile organic compounds (VOCs), ethanol is one of the vital industrial chemical reagents owing to its regular use in numerous areas such as alcoholic beverages, cosmetics, pharmaceuticals, food, paints, and chemical products [38,39]. The direct and constant contact with ethanol vapor, especially at high concentrations, can prompt the irritation of the mucous membranes, nose, and eyes, and can lead to nausea, headaches, and vertigo [39]. Moreover, many traffic accidents can be correlated to the high consumption of alcoholic beverages before driving [40]. As a result, the design of low-cost ethanol gas sensors with effective and selective performance is a critical issue. In recent decades, several kinds of ethanol gas sensors have been studied, like resistive sensors [41] and surface acoustic wave sensors [42]. Metal oxides semiconductors such as ZnO, SnO_2_, and TiO_2_ have garnered much interest as resistive gas sensing materials due to their low cost, easy synthesis, high sensitivity, and simplicity of use. Nechita et al. [43] investigated the gas sensing properties of Nb-doped TiO_2_ porous thin films for ethanol and methanol detection and found that the maximum sensitivity was realized at 367 °C. W/TiO_2_ thin films synthesized by a modified sol–gel technique displayed a high sensitivity to ethanol gas at 350 °C [44]. Phanichphant et al. [45] reported that flame-generated Nb-Doped TiO_2_ had a high sensitivity to ethanol gas at an operating temperature of 400 °C.

Based on our search, there is no available work on the impact of (Gd, Nb) and (Gd, Mo) codoping on the photodegradation of TiO_2_ for reactive yellow 145, brilliant green, and amoxicillin contaminants, as well as for sensing volatile organic compounds. Gd, Nb, and Mo dopants have an electronic configuration including d- and f-orbitals, and can also adopt variable oxidation states that can improve the optical, photocatalytic, and sensing properties of TiO_2_. In this study, new compositions composed of Ti_0.96_Gd_0.01_Nb_0.03_O_2_ and Ti_0.96_Gd_0.01_Mo_0.03_O_2_ plus pure TiO_2_ were synthesized for the depollution of reactive yellow 145, brilliant green, and amoxicillin contaminants under visible light irradiation. These samples were also tested for selective detection of ethanol gas.

## 2. Results and Discussion

### 2.1. X-ray Diffraction (XRD)

**Figure 1:**
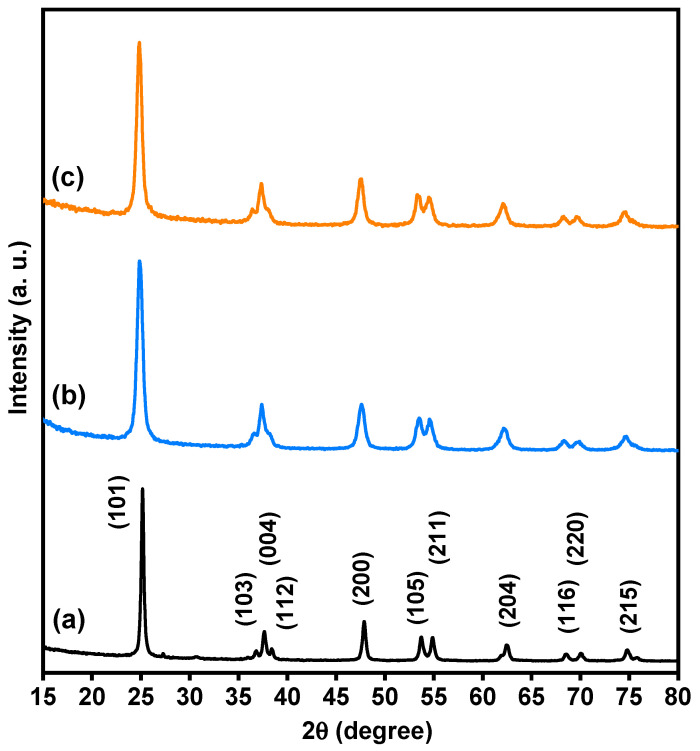
XRD patterns of (**a**) pure TiO_2_, (**b**) Ti_0.96_Gd_0.01_Nb_0.03_O_2_, and (**c**) Ti_0.96_Gd_0.01_Mo_0.03_O_2_ powders.

**Figure 2:**
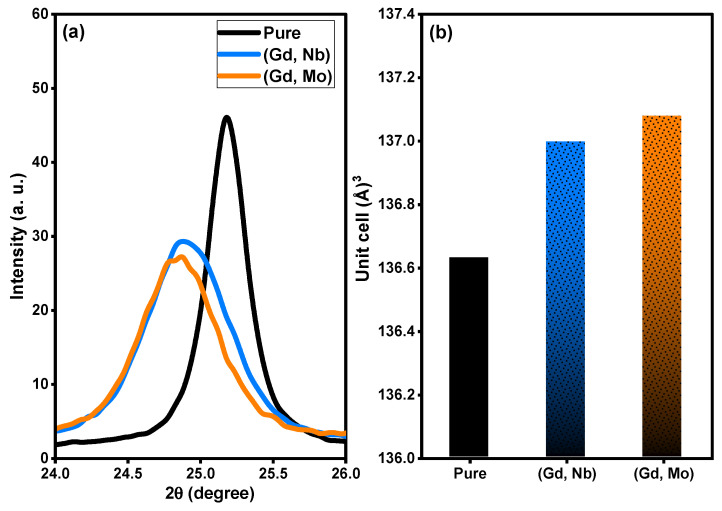
Depicts (**a**) enlargement pattern of (101) plane and (**b**) unit cell volume of pure, (Gd, Nb), and (Gd, Mo) codoped TiO_2_.

Based on XRD analysis, the purity, phases, and crystalline structure of TiO_2_, Ti_0.96_Gd_0.01_Nb_0.03_O_2_ and Ti_0.96_Gd_0.01_Mo_0.03_O_2_ powders were analyzed and are presented in Figure 1. With respect to pure TiO_2_, 11 X-ray diffraction peaks at 2-theta angles of 25.171°, 36.811°, 37.652°, 38.399°, 47.901°, 53.699°, 54.859°, 62.512°, 68.498°, 70.042°, and 74.902° were identified. These diffraction peaks were indexed to the (101), (103), (004), (112), (200), (105), (211), (204), (116), (220), and (215) planes of titanium oxide structure with anatase phase (JCPDS, No. 21-1272). In addition, the two codoped samples had diffraction patterns similar to that of pure TiO_2_, proving the formation of anatase phase. As depicted in Figure 2a, the enlargement pattern of the main XRD peak (101) of TiO_2_ shows shifts to low 2-theta angles after codoping with (Gd, Nb) or (Gd, Mo) ions. These shifts can be attributed to the replacement of Ti^4+^ sites, which have an ionic radius of 0.605 Å (VI), by Gd^3+^ (0.938 Å), Nb^5+^ (0.64 Å) or Mo^6+^ (0.59 Å, VI) ions with a higher ionic radius. In the same context, the unit cell volume of pure TiO_2_ structure was expanded after insertion of (Gd, Nb) and (Gd, Mo) ions into TiO_2_, indicating the actual codoping process as shown in Figure 2b, which was in agreement with many previous studies on Gd, Nb, or Mo-doped TiO_2_ [46,47,48]. The crystallite size of TiO_2_, Ti_0.96_Gd_0.01_Nb_0.03_O_2_ and Ti_0.96_Gd_0.01_Mo_0.03_O_2_ catalysts were considered according to Scherrer-equation [10]:

(1) D=Kλ/βcosθ

**Figure 3:**
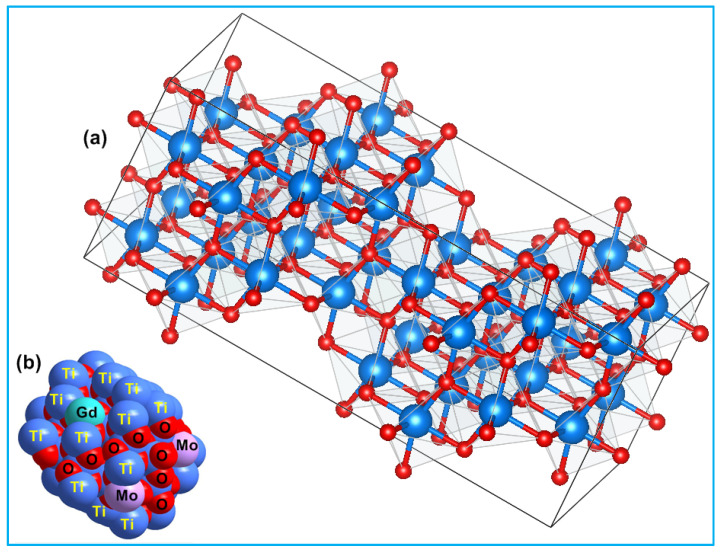
Depicts (**a**) 2 × 2 × 2 primitive cell of anatase TiO_2_ and (**b**) space-filling model of (Gd, Mo) codoped TiO_2_ structure.

The average crystallite size of TiO_2_, Ti_0.96_Gd_0.01_Nb_0.03_O_2_, and Ti_0.96_Gd_0.01_Mo_0.03_O_2_ powders was 27, 14, and 15 nm, respectively. Figure 3 displays the geometrical structure of the primitive unit cell and the space-filling model of anatase TiO_2_ with substitutions by Gd^3+^ and Mo^6+^ ions, illustrating the coordination of the cations and anions.

### 2.2. Band Gap and Optical Features

**Figure 4:**
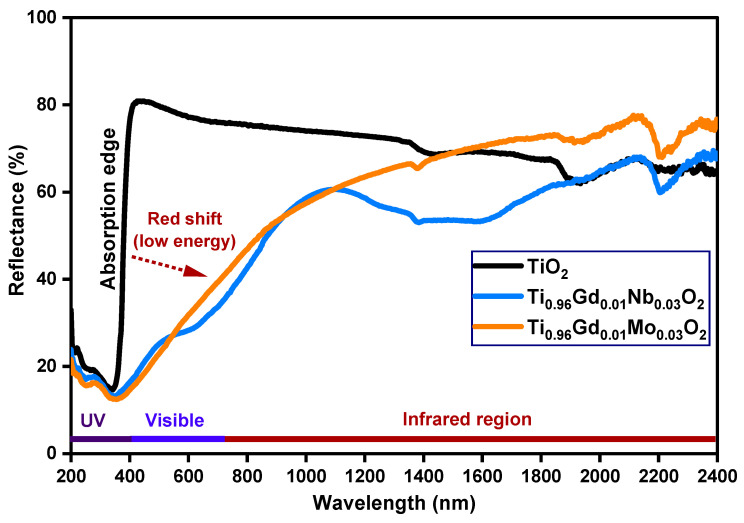
Diffuse reflectance (%) spectra of TiO_2_, Ti_0.96_Gd_0.01_Nb_0.03_O_2_, and Ti_0.96_Gd_0.01_Mo_0.03_O_2_ samples.

**Figure 5:**
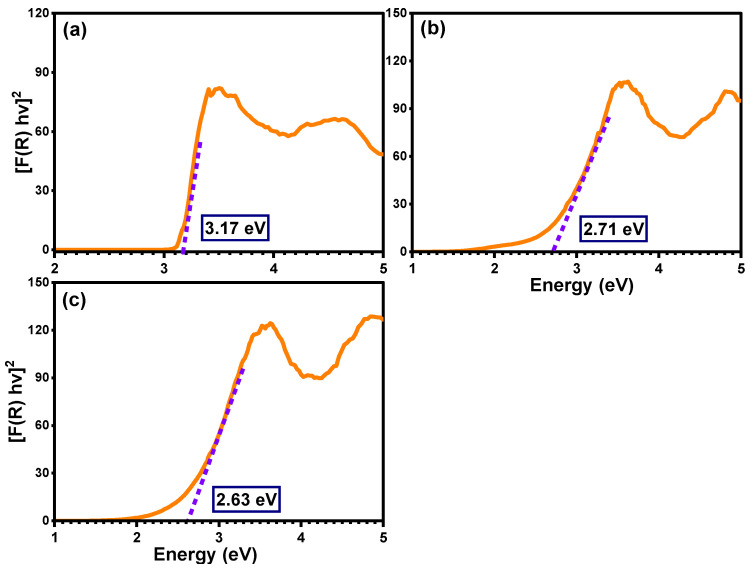
Band gap estimation for (**a**) pure TiO_2_, (**b**) Ti_0.96_Gd_0.01_Nb_0.03_O_2_, and (**c**) Ti_0.96_Gd_0.01_Mo_0.03_O_2_ samples.

The detailed optical properties of pure TiO_2_, Ti_0.96_Gd_0.01_Nb_0.03_O_2_, and Ti_0.96_Gd_0.01_Mo_0.03_O_2_ samples were identified by diffuse reflectance (DR), analysis as demonstrated in Figure 4. The diffuse reflectance spectrum of the pure TiO_2_ sample shows a large and sharp decrease in intensity below 400 nm (ultraviolet region), corresponding to the band gap energy. After the insertion of (Gd, Nb) and (Gd, Mo) ions into the TiO_2_ semiconductor, the absorption edge was strongly red-shifted (to a lower energy), implying that the electronic structure of the pure anatase TiO_2_ was altered by codoping with (Gd, Nb) and (Gd, Mo) ions. The Tauc equation and Kubelka–Munk formula were applied to estimate the value of the band gap energy of pure TiO_2_, Ti_0.96_Gd_0.01_Nb_0.03_O_2_, and Ti_0.96_Gd_0.01_Mo_0.03_O_2_, as indicated below [49]:

(2) $$\alpha =A\left(h\nu -Eg\right)n/h\nu$$

(3) $$F\left(R\right)=(1-R{)}^{2}/2R$$

where α symbolizes the absorption coefficient, hν represents the energy of the photon, Eg means the band gap energy, F(R) indicates the Kubelka–Munk function, R designates the reflectance, and S represents the scattering coefficient. As demonstrated in Figure 5, the plot of [F(R) hν]^2^ on the Y-axis with the photon energy on the X-axis reveals an accurate value for the band gap energy. The detected value of the band gap energy of the pure TiO_2_ semiconductor was 3.17 eV, which was agreement with many previously published studies [50,51]. The band gap energies of the Ti_0.96_Gd_0.01_Nb_0.03_O_2_ and Ti_0.96_Gd_0.01_Mo_0.03_O_2_ compositions were found to be 2.71 and 2.63 eV, respectively. These reductions (red-shifts) of the band gap of the anatase TiO_2_ structure can be correlated to the introduction of impurity states within the band gap structure, as well as the formation of oxygen vacancies for charge neutrality [48,52,53].

### 2.3. Scanning Electron Microscope (SEM)

**Figure 6:**
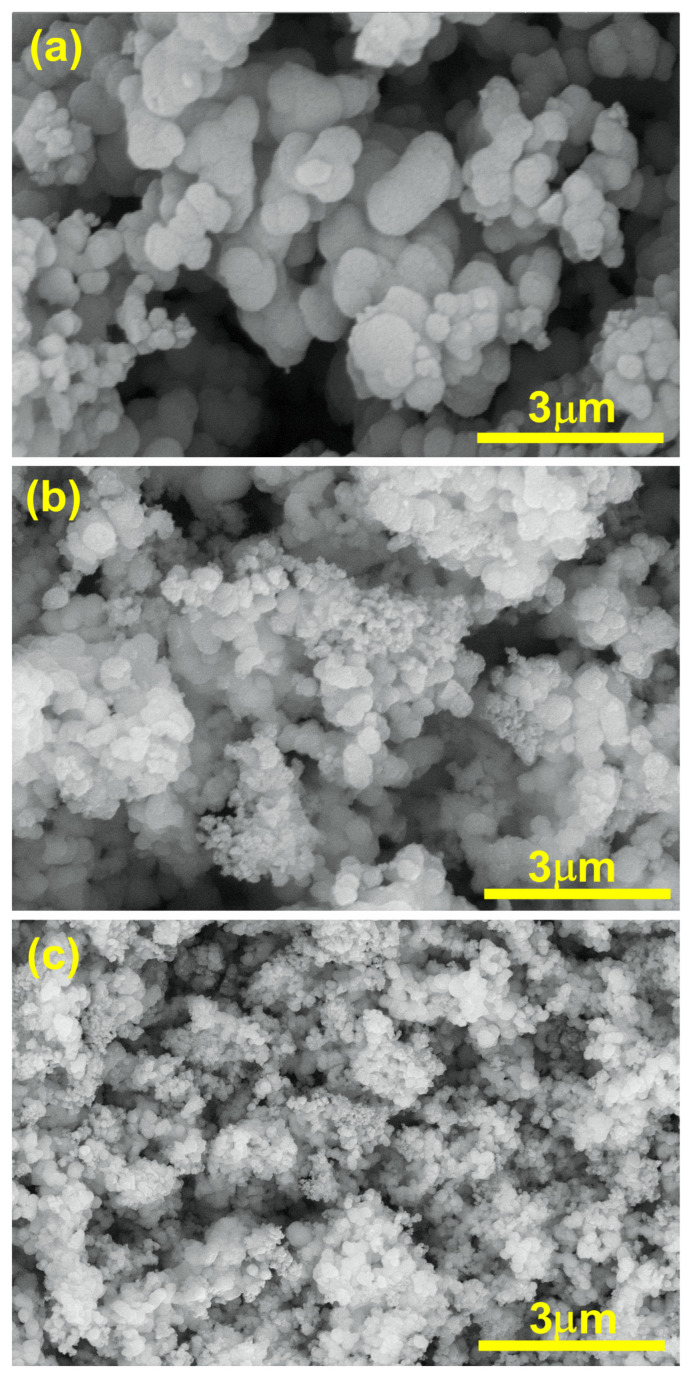
SEM images of (**a**) pure TiO_2_, (**b**) Ti_0.96_Gd_0.01_Nb_0.03_O_2_, and (**c**) Ti_0.96_Gd_0.01_Mo_0.03_O_2_ samples.

**Figure 7:**
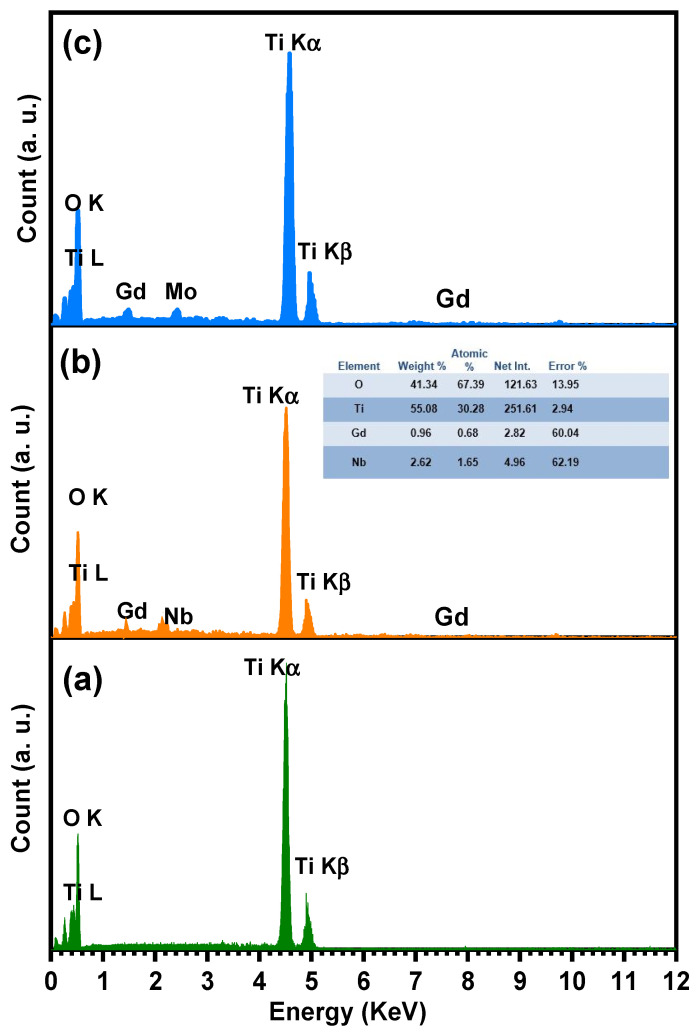
Energy-dispersive X-ray (EDX) spectrum of (**a**) pure TiO_2_, (**b**) Ti_0.96_Gd_0.01_Nb_0.03_O_2_, and (**c**) Ti_0.96_Gd_0.01_Mo_0.03_O_2_ samples.

The SEM analysis of TiO_2_, Ti_0.96_Gd_0.01_Nb_0.03_O_2_, and Ti_0.96_Gd_0.01_Mo_0.03_O_2_ was performed as illustrated in Figure 6. The SEM micrograph of pure TiO_2_ powder reflects the formation of agglomerated grains with nearly spherical structure. The insertion of (Gd, Nb) and (Gd, Mo) ions reduces the grains size of TiO_2_, with the formation of fine particles. The presence of Gd^3+^, Nb^5+^, and Mo^6+^ ions during the calcination step limit the grain growth of TiO_2_ particles. The reduction in grain size has a useful effect on the photocatalytic and gas sensing properties as it offers more surface area and active sites for the reactions. For TiO_2_, Ti_0.96_Gd_0.01_Nb_0.03_O_2_, and Ti_0.96_Gd_0.01_Mo_0.03_O_2_, energy-dispersive X-ray (EDX) examinations were performed to detect the elements present in the samples, as revealed in Figure 7. The spectrum of the EDX analysis of TiO_2_, Ti_0.96_Gd_0.01_Nb_0.03_O_2_, and Ti_0.96_Gd_0.01_Mo_0.03_O_2_ confirms the presence of Ti, O, Gd, Nb, and Mo elements and ruled out the presence of any other contamination. The wt% of the Gd, Nb, and Mo dopants is close to the added weight during the synthesis process.

### 2.4. Depollution of Organic Pollutants

**Figure 8:**
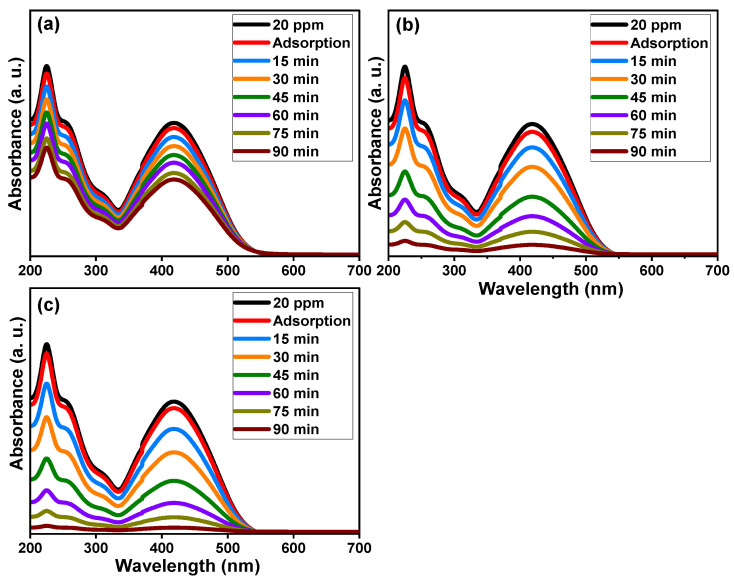
Absorbance performance of reactive yellow 145 dye of (**a**) pure TiO_2_, (**b**) Ti_0.96_Gd_0.01_Nb_0.03_O_2_, and (**c**) Ti_0.96_Gd_0.01_Mo_0.03_O_2_ catalysts under sunlight.

**Figure 9:**
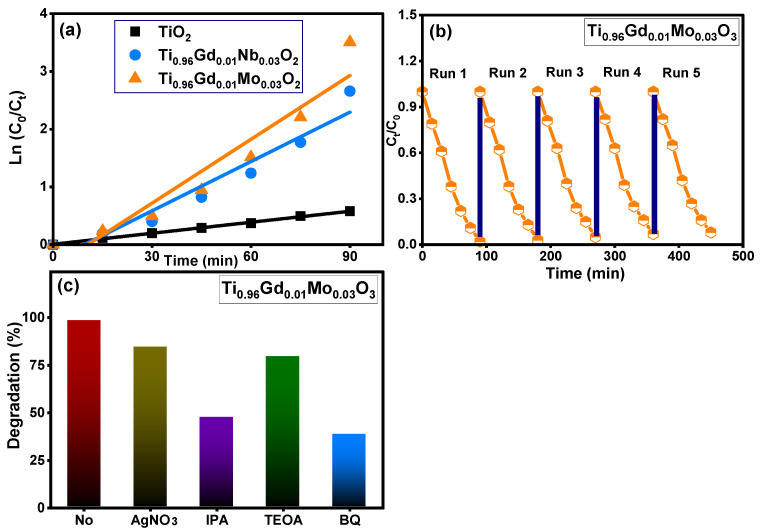
Depicts (**a**): ln (C_0_/C_t_) with time for all samples, (**b**): reusability (**c**): effective radicals test for Ti_0.96_Gd_0.01_Mo_0.03_O_2_ catalyst.

Figure 8 demonstrates the absorbance performance of 20 mg/L reactive yellow 145 solution in the presence of pure TiO_2_, Ti_0.96_Gd_0.01_Nb_0.03_O_2_, and Ti_0.96_Gd_0.01_Mo_0.03_O_2_ catalysts over an irradiation time of 90 min under sunlight energy. Initial adsorption measurements under dark conditions for all samples for 40 min confirm their insignificant effects (3–5%) on the elimination of the reactive yellow dye. Under irradiation, the absorbance curve shows a low decrease in the case of the pure TiO_2_ catalyst, with maximum efficiency of 44% after 90 min. The performance was significantly better for the Ti_0.96_Gd_0.01_Nb_0.03_O_2_ and Ti_0.96_Gd_0.01_Mo_0.03_O_2_ catalysts, with total measured efficacies of 93% and 98% after 90 min of sunlight illumination, respectively. These high activities can be correlated to the broad absorption of visible light. In addition, the Gd^3+^, Nb^5+^, and Mo^6+^ ions can behave as active sites for trapping the electrons, which effectively inhibits charge carrier recombination (electron–hole pairing). As illustrated in Figure 9a, the kinetic plots of the reactive yellow 145 dye in the presence of TiO_2_, Ti_0.96_Gd_0.01_Nb_0.03_O_2_, and Ti_0.96_Gd_0.01_Mo_0.03_O_2_ verify that the reactions follow a linear pseudo-first order with R^2^ values of 0.99, 0.93, and 0.91, with an estimated rate constant equal to 0.0063, 0.028, and 0.037 min^−1^, respectively. The reusability performance was explored for the Ti_0.96_Gd_0.01_Mo_0.03_O_2_ catalyst, as the sample with the best sample activity toward 20 mg/L reactive yellow dye over five cycles, as shown in Figure 9b. After the degradation test, the catalyst was collected by centrifugation and dried at 80 °C to be used for the next degradation experiment. The estimated degradation efficacy was 98, 97, 95, 93, and 92% for five cycles, indicating the effective reuse properties of the Ti_0.96_Gd_0.01_Mo_0.03_O_2_ catalyst. The mechanism of the photodegradation process is mainly related to the active radicals; to detect these radicals, four different chemical agents [54] involving triethanolamine (TEOA), benzoquinone (BQ), AgNO_3_ and isopropyl alcohol (IPA) were used to attack the holes (h^+^), superoxide (O_2_^−^), electrons (e^−^), and hydroxyl (˙OH) species, respectively. The obtained results, presented in Figure 9c, show that the photocatalytic activity of Ti_0.96_Gd_0.01_Mo_0.03_O_2_ catalyst towards reactive yellow 145 dye was noticeably reduced after the addition of BQ and IPA, which confirmed that the O_2_^−^ and ˙OH species were the fundamentals radicals in the depollution process.

**Figure 10:**
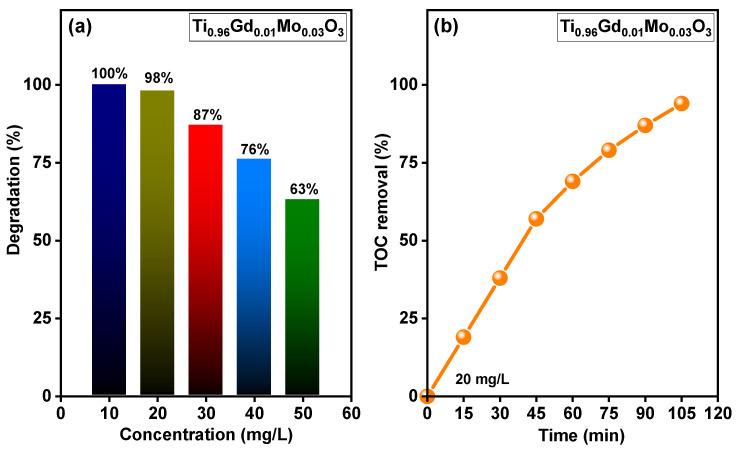
Displays (**a**) effect of concentration of reactive yellow 145 on the photodegradation efficiency of Ti_0.96_Gd_0.01_Mo_0.03_O_2_ catalyst and (**b**) TOC removal rate of reactive yellow 145 over time for Ti_0.96_Gd_0.01_Mo_0.03_O_2_ catalyst.

The relation between the concentration of the reactive yellow 145 dye and the photodegradation efficiency of Ti_0.96_Gd_0.01_Mo_0.03_O_2_ catalyst was shown in Figure 10a. At dye concentration of 10 mg/L, Ti_0.96_Gd_0.01_Mo_0.03_O_2_ catalyst reveals a photodegradation activity of ~100 after 40 min of sunlight irradiation while at 20 mg/L it exhibits a photodegradation efficiency of 98% after 90 min. In case concentration of 30, 40 and 50 mg/L, this catalyst has photodegradation efficiencies of 87, 76 and 63% after 90 min, respectively. Figure 10b shows the outcomes of the total organic carbon (TOC) study of reactive yellow 145 dye for Ti_0.96_Gd_0.01_Mo_0.03_O_2_ catalyst. The TOC examination was performed to study the mineralization performance of reactive yellow 145 dye via the photocatalytic process. The achieved findings verify that the rate of TOC removal increased with the irradiation time, reaching 95% after 105 min, indicating the full photodegradation of reactive yellow 145 dye by the Ti_0.96_Gd_0.01_Mo_0.03_O_2_ catalyst.

**Figure 11:**
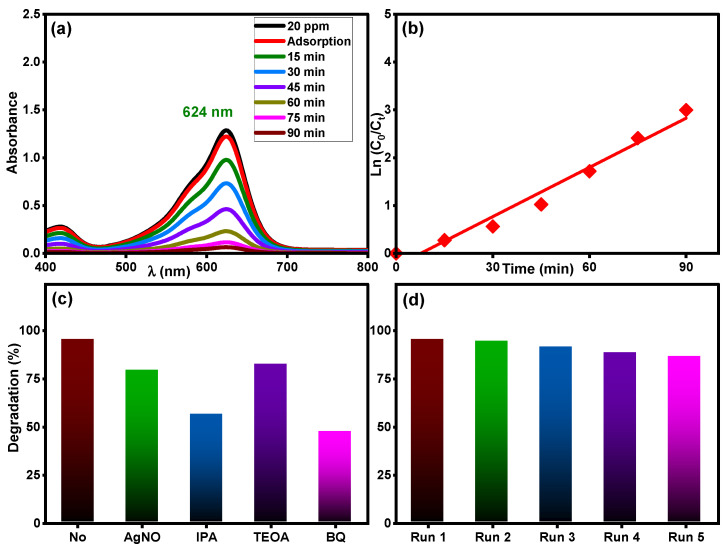
(**a**) Absorbance performance of 20 mg/L brilliant green dye, (**b**) ln (C_0_/C_t_) over time, (**c**) effective radicals test, and (**d**) reusability of Ti_0.96_Gd_0.01_Mo_0.03_O_2_ catalyst.

The photocatalytic activity of the Ti_0.96_Gd_0.01_Mo_0.03_O_2_ catalyst was tested for another organic pollutant, brilliant green dye at concentration of 20 mg/L under sunlight irradiation. As demonstrated in Figure 11a, the maximum absorbance peak of brilliant green dye at 624 nm was efficiently decreased over time, with a final efficiency of 95% after 90 min. The kinetic plot of brilliant green dye degradation, Figure 11b, confirms the pseudo-first-order behavior (R^2^ = 0.97) with a measured rate constant of 0.0343 min^−1^. As demonstrated in Figure 11c, the Ti_0.96_Gd_0.01_Mo_0.03_O_2_ catalyst exhibited a high reusability for brilliant green dye, with efficiency of 95, 94, 91, 88, and 86% for five repeated tests, respectively. The scavenger tests reveal that O_2_˙^−^ and ˙OH radicals were essential for the degradation reaction of brilliant green dye, as shown in Figure 11d.

**Figure 12:**
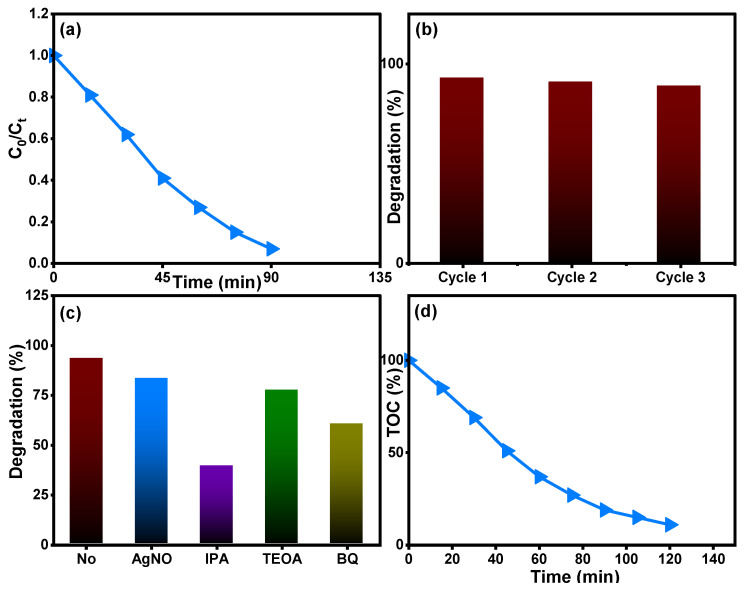
Describes (**a**) (C_t_/C_0_) over time, (**b**) reusability, (**c**) scavenger tests, and (**d**) TOC analysis of Ti_0.96_Gd_0.01_Mo_0.03_O_2_ catalyst for degradation of the antiobiotic amoxicillin.

Figure 12 depicts the removal properties of the Ti_0.96_Gd_0.01_Mo_0.03_O_2_ catalyst for 20 mg/L amoxicillin antibiotic. The relation between C_0_/C_t_ over time shows that the Ti_0.96_Gd_0.01_Mo_0.03_O_2_ catalyst has a photocatalytic activity of 93% with an irradiation time of 90 min (Figure 12a). The recyclability experiment for the Ti_0.96_Gd_0.01_Mo_0.03_O_2_ catalyst with 20 mg/L amoxicillin confirmed its high degradation efficiency over three cycles (Figure 12b). The scavenger experiments revealed that ˙OH was the main radicals in the degradation reaction, followed by a moderate effect of O_2_˙^−^ species, as depicted in Figure 12c. The total organic carbon analysis, Figure 12d, shows the effective mineralization of amoxicillin to CO_2_ and H_2_O with an efficiency of 87% after 120 min. The photodegradation or mineralization reaction for the used organic pollutants is basically related to the photo-generation of electrons from the valence band (VB) to the conduction band (CB) by light photons. These excited electrons can attack the oxygen molecules present in the waste solution to produce the O_2_˙^−^ species while the holes (h^+^) connect to the water molecules to produce the ˙OH species. The active reaction occurring between mainly ˙OH and O_2_˙^−^ radicals and reactive yellow 145, brilliant green, and amoxicillin contaminants yields CO_2_ and H_2_O, as shown below [22,25,28]:

Ti_0.96_Gd_0.01_Mo_0.03_O_2_ → visible light–UV photons → e^−^ + h^+^ pairs

Gd^3+^ + e^−^ → Gd^2+^

Mo^6+^ + e^−^ → Mo^5+^

Gd^2+^, Mo^5+^ or e^−^ + O_2_ → O_2_^−^

h^+^ + H_2_O → ˙OH + H^+^

(OH˙, O_2_˙^−^) → Reactive yellow 145, brilliant green, and amoxicillin → CO_2_ and H_2_O

### 2.5. Gas Sensing Properties

**Figure 13:**
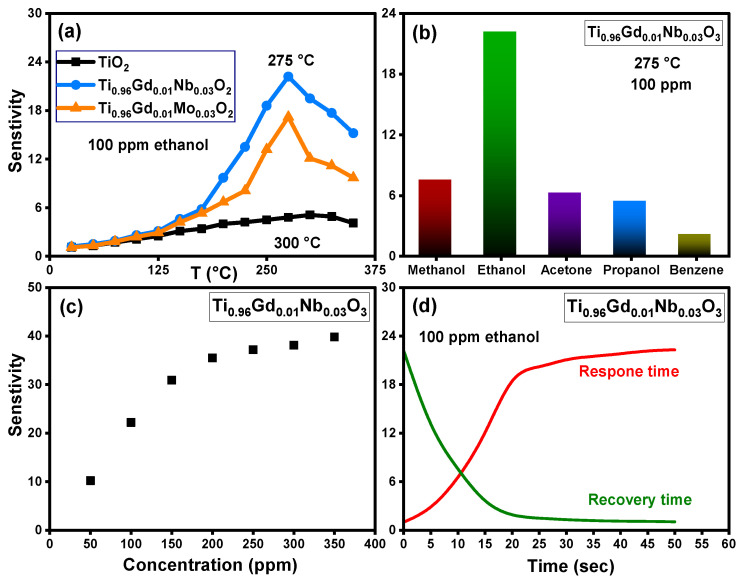
Illustrating (**a**) sensitivity with temperature for 100 ppm ethanol gas of pure TiO_2_, Ti_0.96_Gd_0.01_Nb_0.03_O_2_, and Ti_0.96_Gd_0.01_Mo_0.03_O_2_ sensors, (**b**) sensitivity of Ti_0.96_Gd_0.01_Nb_0.03_O_2_ sensor towards different gases, (**c**) sensitivity–concentration curve, and (**d**) response and recovery times of 100 ppm ethanol.

The sensing response of pure TiO_2_, Ti_0.96_Gd_0.01_Nb_0.03_O_2_, and Ti_0.96_Gd_0.01_Mo_0.03_O_2_ sensors toward different volatile organic compounds was tested at different temperatures. Figure 13a shows the relation between the sensitivity and temperature of pure TiO_2_, Ti_0.96_Gd_0.01_Nb_0.03_O_2_, and Ti_0.96_Gd_0.01_Mo_0.03_O_2_ sensors for ethanol gas at a concentration of 100 ppm. The temperature-sensitive performance of these samples clearly show that the sensitivity slowly increased with temperature for pure TiO_2_ and attained the maximum response of 5.1 at 300 °C. The gas sensing performance of Ti_0.96_Gd_0.01_Nb_0.03_O_2_ and Ti_0.96_Gd_0.01_Mo_0.03_O_2_ sensors presented enhanced response to ethanol gas, with a recorded maximum sensitivity value of 22.2 and 17.2 at an operating temperature of 275 °C, respectively. The addition of (Gd, Nb) ions enhances both the response value and the operating temperature. It appears that the existence of (Gd, Nb) ions enhances the reaction between the sensor surface and ethanol gas by improving the active sites and activation energy due to the reduced grain size. The selectivity of the Ti_0.96_Gd_0.01_Mo_0.03_O_2_ sensor was examined for different volatile organic compounds at a temperature of 275 °C and with equivalent concentrations of 100 ppm for all gases, as shown in Figure 13b. The results of this measurement indicate that the response values were 7.5, 22.2, 6.2, 5.4, and 2.1 for methanol, ethanol, acetone, propanol, and benzene, respectively. As a result, the Ti_0.96_Gd_0.01_Mo_0.03_O_2_ sensor possesses a good and selective response performance at 275 °C for ethanol gas. Figure 13c depicts the relation between the sensitivity and ethanol gas concentration detected by the Ti_0.96_Gd_0.01_Mo_0.03_O_2_ sensor at a working temperature of 275 °C. Linear performance of the sensitivity can be seen within the concentration range of 50–200 ppm; above this value, the sensitivity increased slowly. The response and recovery times of the sensor are essential parameters for practical use [55,56]. As demonstrated in Figure 13d, the Ti_0.96_Gd_0.01_Mo_0.03_O_2_ sensor has a response time nearly equal to 20 s and a recovery time of 15 s for 100 ppm ethanol gas. The mechanism of response to ethanol gas for the Ti_0.96_Gd_0.01_Mo_0.03_O_2_ sensor is principally related to the reaction between the adsorbed oxygen ions on sensor surface (O^–^, O^2−^) and the target gas (ethanol). At higher temperatures the adsorbed oxygen (O_2_) on the Ti_0.96_Gd_0.01_Mo_0.03_O_2_ sensor material changes to O^–^ or O^2−^ ions by capturing electrons, and the resistance increases [57,58]. After injection, the ethanol gas reacts with the O^−^ or O^2−^ ions on surface to liberate the electrons on the sensor, which reduces the resistance, leading to a high response. It seems that the presence of Gd^3+^ and Nb^5+^ ions facilitate this reaction, which enhances the sensitivity of Ti_0.96_Gd_0.01_Mo_0.03_O_2_ sensor. In this study, the results show that (Gd, Mo) is more effective for inducing the photocatalytic depollution properties of the TiO_2_ semiconductor while (Gd, Nb) is the best blend to reinforce its selective sensing performance towards ethanol vapor.

## 3. Experimental

### 3.1. Synthesis of TiO_2_-Based Compositions

Titanium butoxide (Ti(OBu)₄, 98%), gadolinium nitrate (Gd(NO_3_)_3_, 99.9%), niobium pentachloride (NbCl_5_, 99%), molybdenum chloride (MoCl_5_, 99.9%), and ammonium hydroxide (NH_4_OH) were used as starting materials to synthesize pure, (Gd, Nb) and (Gd, Mo) codoped TiO_2_ compositions. For pure TiO_2_, 12.8 mL Ti(C_4_H_9_O)_4_ reagent was dissolved in 40 mL butanol with constant stirring for 0.5 h. Then, ammonium hydroxide (NH_4_OH, 33%) was dropped into the solution until a pH of 7.8 was reached, to yield the precipitate. To remove any contaminants or impurities, the obtained precipitate was washed by deionized water several times and then dried at 105 °C. In an electric furnace, the obtained powder was placed into crucible and calcined at 610 °C for 3.5 h. For (Gd, Nb) and (Gd, Mo) codoped TiO_2_ with compositions of Ti_0.96_Gd_0.01_Nb_0.03_O_2_ and Ti_0.96_Gd_0.01_Mo_0.03_O_2_, the same steps were applied, similar to the pure TiO_2_ sample, but NbCl_5_ and MoCl_5_ were dissolved into Ti(OBu)₄ solution before precipitation, as demonstrated in Table 1.

### 3.2. Characterization and Measurements

X-ray diffraction (PANalytical), scanning electron microscope (Quanta 250), and diffuse reflectance JASCO spectrophotometer (V-570) techniques were used to characterize the synthesized powders. The Rietveld refinement method using Match Crystal Impact (version 3.15 Build 278) linked with FullProf software Suite (January2018_XP) was used to estimate the lattice parameter and unit cell volume of the samples. The photocatalytic degradation features of pure TiO_2_, Ti_0.96_Gd_0.01_Nb_0.03_O_2_, and Ti_0.96_Gd_0.01_Mo_0.03_O_2_ catalysts were measured against 20 mg/L reactive yellow 145 and 20 mg/L brilliant green dyes as well as 20 mg/L amoxicillin antibiotic under natural sunlight. First, 50 mg of TiO_2_, Ti_0.96_Gd_0.01_Nb_0.03_O_2_, or Ti_0.96_Gd_0.01_Mo_0.03_O_2_ catalyst was added to a beaker containing 100 mL of dye or antibiotic solution in the dark for 40 min (with constant stirring) to evaluate the adsorption factor and then the solution was placed in sunlight (July, 1–3 pm). Every 15 min, 4 mL of the irradiated solution was collected and centrifuged (11,000 rpm) to remove the particles of the powder. The photocatalytic degradation efficiency was determined by measuring the changes in the absorbance of the maximum peak of reactive yellow 145 (418 nm), brilliant green (624), and amoxicillin antibiotic (270 nm) over time. The photodegradation efficiency (PE) is expressed as follows:

(4) $$PE=\frac{A_{t}}{A_{0}}=\frac{C_{t}}{C_{o}}$$

where A_0_ and C_0_ mean the principal absorbance and concentration of reactive yellow 145, brilliant green, and amoxicillin, whereas A_t_ and C_t_ specify to the absorbance and concentration of reactive yellow 145, brilliant green, and amoxicillin during the different irradiation times. The measurements of the gas sensing properties of pure TiO_2_, Ti_0.96_Gd_0.01_Nb_0.03_O_2_, and Ti_0.96_Gd_0.01_Mo_0.03_O_2_ sensors in pellet form were carried out in a homemade gas chamber. Various gases (methanol, ethanol, propanol, acetone, and benzene) were inserted into the chamber by micro-syringe. The resistivity of pure TiO_2_, Ti_0.96_Gd_0.01_Nb_0.03_O_2_, and Ti_0.96_Gd_0.01_Mo_0.03_O_2_ gas sensors was measured by a Keithley device (model 2450) at different temperatures and the sensitivity value was calculated based on the formula:

(5) $$Sensitivity\left(S\right)=\frac{R_{a}}{R_{g}}$$

where R_a_ is the resistance of TiO_2_, Ti_0.96_Gd_0.01_Nb_0.03_O_2_, and Ti_0.96_Gd_0.01_Mo_0.03_O_2_ sensors in air; and R_g_ is the resistance of TiO_2_, Ti_0.96_Gd_0.01_Nb_0.03_O_2_, and Ti_0.96_Gd_0.01_Mo_0.03_O_2_ sensors in the different gases.

## Figures and Tables

**Table 1:** Detailed weights of chemical regents used in synthesis of pure TiO_2_, Ti_0.96_Gd_0.01_Nb_0.03_O_2_, and Ti_0.96_Gd_0.01_Mo_0.03_O_2_ powders.

Composition	Ti(OBu)₄ (mL)	Gd(NO_3_)_3_ (g)	NbCl_5_ (g)	MoCl_5_ (g)
TiO_2_	12.8	-	-	-
(1% Gd, 3% Nb)	12.276	0.1287	0.304	-
(1% Gd, 3% Mo)	12.276	0.1287	-	0.307

## Data Availability

The datasets are available on reasonable request.
